# Rudolf Virchow Fund

**Published:** 1901-05

**Authors:** Charles A. L. Reed, Henry P. Bowditch, William K. Welch, Robert F. Weir, A. Jacobi

**Affiliations:** President of the American Medical Association; President of the Congress of American Physicians and Surgeons; Johns Hopkins University; President of the New York Academy of Medicine; 110 West 34th Street, New York, Secretary


### RUDOLF VIRCHOW FUND.

*To the American Medical Profession:* On October 13, 1901, *Rudolf Virchow* will be eighty years old. When he completed his seventieth year a fund was started in his honor to enable the great master to facilitate scientific research by establishing scholarships, and by encouraging special medical and biological studies. Contributions to that *"Rudolf Virchow Fund"* were furnished by those in all countries interested in progressive medicine, as a homage to the man whose name is always certain to arouse admiration and enthusiasm.

In Berlin a large committee, containing amongst others the names of A. Bastian, V. Coler, A. Entenburg, B. Fraenkel, O. Israel, Fr. Koenig, C. Posner and W. Waldeyer, has been formed to call for contributions which are to be added to the original *"Rudolf*

*Virchow Fund,"* so as to increase its efficiency. The committee expresses the opinion that in no better way, and in none more agreeable to the great leader of modern medicine, can his eightieth birthday be celebrated, and asks for the sympathy and cooperation of all those engaged in the study and practice of scientific medicine all over the globe.

The undersigned have formed a sub-committee for the purpose of making the American profession acquainted with the intentions of the Berlin committee, and urge their colleagues to participate in honoring the very man who has done more these fifty years than any other to make medicine a science, and international.

Subscriptions should be sent to their secretary, who will receipt therefor.

CHARLES A. L. REED,
President of the American Medical Association.

HENRY P. BOWDITCH,
President of the Congress of American Physicians and Surgeons.

WILLIAM K. WELCH,
Johns Hopkins University.

ROBERT F. WEIR,
President of the New York Academy of Medicine.

A. JACOBI,
110 West 34th Street, New York, Secretary.

